# Dr. Simmons and the Octopus Again

**Published:** 1905-12

**Authors:** 


### DR. SIMMONS AND THE OCTOPUS AGAIN.

I have a letter from one of the best known medical editors in the South, from which I extract the following. He wants information, and I give it:

"Why those in authority in the Americal Medical Association ever made him [Simmons] editor of the journal, I never could understand. It might be accounted for in this way: A man thoroughly capable of editing and managing the *Journal of the A. M. A.* would have to make a great sacrifice, financially, to accept the position. To make a success of the Association, they ought to pay a $10,000 salary, and *remove Dr. Simmons immediately.* As long as they pay only $5000 a year they can not expect to get a very high-class man for the position."

It was a put up job; a cut and dried ring job for Simmons, all *American competition* for the position being prevented by a ruling of the Board of Trustees, that the editor *should not engage in practice,* or any other kind of work. Of course, no capable man could afford to give up his practice for a $5000 salary, and this effectually closed the doors and narrowed the selection down to a class of men who *could do so,* or who had no practice to give up. I say "American competition"; Dr. Simmons is not a native American; he is a foreigner; McCormack says he is an Englishman. My information from Chicago is that he is an Irishman, and was an attache in some capacity of Dublin Castle. I presume he has taken out naturalization papers and can vote. He was a homeopathic practitioner from 1882 to 1894, when he received a diploma from Rush. Father Davis, who founded the *Journal* and gave it a high character, did a large practice and was teacher in the Northwestern Medical College. The great Hamilton, who succeeded him and wore his mantle with honor and renown was, in addition to being editor of the *Journal,* Professor of Surgery in Rush; Superintendent State Insane Asylum; Surgeon M. H. S.; President of the Illinois Historical Society, and besides did a large general practice. It was not required of them to give up practice. The place was "fixed" for Simmons by one Priestly, so I am informed by a Chicago physician of world-wide celebrity.

